# Fabrication of Antibacterial TiO_2_ Nanostructured Surfaces Using the Hydrothermal Method

**DOI:** 10.1021/acsomega.2c06175

**Published:** 2022-12-07

**Authors:** Niharika Rawat, Metka Benčina, Ekaterina Gongadze, Ita Junkar, Aleš Iglič

**Affiliations:** † Laboratory of Physics, Faculty of Electrical Engineering, University of Ljubljana, Tržaška 25, SI-1000 Ljubljana, Slovenia; ‡ Department of Surface Engineering, Jožef Stefan Institute, Jamova 39, SI-1000 Ljubljana, Slovenia; § Laboratory of Clinical Biophysics, Faculty of Health Sciences, University of Ljubljana, Zdravstvena pot 5, SI-1000 Ljubljana, Slovenia; ∥ Chair of Orthopaedic Surgery, Faculty of Medicine, University of Ljubljana, Vrazov trg 2, SI-1000 Ljubljana, Slovenia

## Abstract

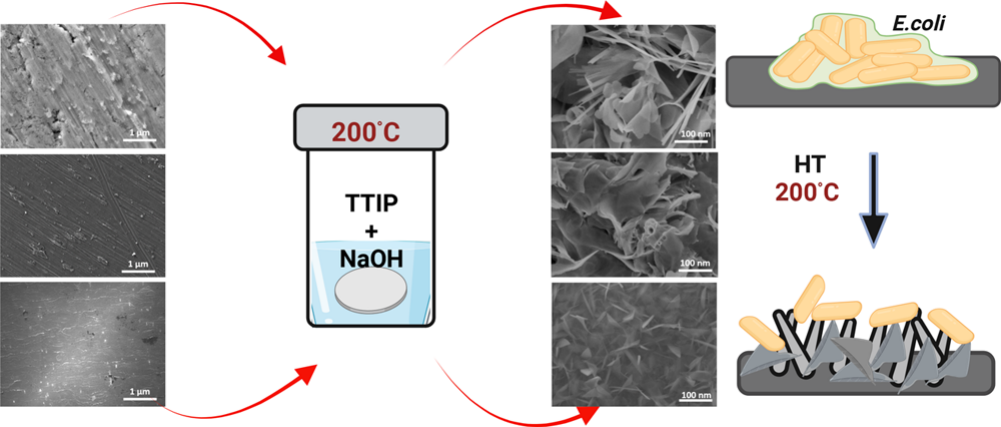

Implant-associated infections (IAI) are a common cause for implant failure, increased medical costs, and critical for patient healthcare. Infections are a result of bacterial colonization, which leads to biofilm formation on the implant surface. Nanostructured surfaces have been shown to have the potential to inhibit bacterial adhesion mainly due to antibacterial efficacy of their unique surface nanotopography. The change in topography affects the physicochemical properties of their surface such as surface chemistry, morphology, wettability, surface charge, and even electric field which influences the biological response. In this study, a conventional and cost-effective hydrothermal method was used to fabricate nanoscale protrusions of various dimensions on the surface of Ti, Ti_6_Al_4_V, and NiTi materials, commonly used in biomedical applications. The morphology, surface chemistry, and wettability were analyzed using scanning electron microscopy (SEM), X-ray photoemission spectroscopy (XPS), and water contact angle analysis. The antibacterial efficacy of the synthesized nanostructures was analyzed by the use of *Escherichia coli* bacterial strain. XPS analysis revealed that the concentration of oxygen and titanium increased on Ti and Ti_6_Al_4_V, which indicates that TiO_2_ is formed on the surface. The concentration of oxygen and titanium however decreased on the NiTi surface after hydrothermal treatment, and also a small amount of Ni was detected. SEM analysis showed that by hydrothermal treatment alterations in the surface topography of the TiO_2_ layer could be achieved. The oxide layer on the NiTi prepared by the hydrothermal method contains a low amount of Ni (2.8 atom %), which is especially important for implantable materials. The results revealed that nanostructured surfaces significantly reduced bacterial adhesion on the Ti, Ti_6_Al_4_V, and NiTi surface compared to the untreated surfaces used as a control. Furthermore, two sterilization techniques were also studied to evaluate the stability of the nanostructure and its influence on the antibacterial activity. Sterilization with UV light seems to more efficiently inhibit bacterial growth on the hydrothermally modified Ti_6_Al_4_V surface, which was further reduced for hydrothermally treated Ti and NiTi. The developed nanostructured surfaces of Ti and its alloys can pave a way for the fabrication of antibacterial surfaces that reduce the likelihood of IAI.

## 1 Introduction

Titanium (Ti) and its alloys are widely used in the biomedical field for implant application due to their biocompatibility, corrosion resistance, and mechanical strength, which is close to that of the bone.^1^ Low Young’s modulus of Ti and its alloys offers a biomechanical advantage due to lower stress shielding that provides efficient bone regeneration compared to other implant materials.^2^ In addition to artificial bones, joint replacement, and dental implants, titanium and its alloys are also used for cardiovascular implants as vascular stents, pacemakers, circulatory devices, etc. Due to their inert, non-magnetic, and strong properties, alloys like Nitinol (NiTi) have garnered significant attention as an eminent diagnostic tool such as magnetic resonance imaging.^3^ NiTi is commonly used for stents to treat cardiovascular diseases; in many cases, NiTi is coated, usually with a thin carbon film to improve blood compatibility.^4^ Ti and its alloys are known to naturally form a highly adherent and chemically stable protective oxide layer on their surface which improves their corrosion resistance.^5^ The thickness and composition of this protective oxide layer (usually TiO_2_) depends on the surrounding environmental conditions.^6^ Favorable properties of Ti and its alloys make them suitable for total joint replacement surgeries; however, implant failure presents a prevalent and implacable threat. About 1.5–2.5% of orthopedic implants turn into the site of infection due to bacterial contamination causing implant associated infections (IAI) which are the primary reason for implant failure.^7^ IAI have attracted considerable attention over the years to succumb to elevating postsurgical medical costs. As implants are exposed to a harsh biological environment, biofilm formation and implant failure are often inevitable. IAI lead to severe morbidity, postsurgical complications, an increase in medical costs, and mortality rate between 2.7 and 18%.^8,9^ The fate of implant materials is not only governed by the bulk material but also by the various surface properties such as roughness, morphology, electrical properties, composition, and hydrophilicity/hydrophobicity.

Therefore, considerable attention has been focused on the modification of implant biomaterial surfaces, especially surface chemistry.^10^ Various surface modification approaches have been proposed to enhance the biocompatibility of implant surfaces, such as antibacterial loaded coatings, electropolishing procedures, bioceramic coatings, polymeric coatings,^11^ and sol–gel method.^12,13^ These approaches aim to alter the physicochemical properties of the surface, which further dictates interaction with biological materials.^14,15^ Modification of surface morphology to nanoranges may inhibit the bacterial attachment to the surface due to the limited surface-to-volume ratio. Through modification of topography, different types of nanoprotrusions can be generated on the surface. Mechanical interaction of nanoprotrusions with bacteria leads to penetration of bacterial cell membranes and results in oxidative stress and cell death.^16^ Ivanova and co-workers^17^ studied the bactericidal effect of nanoprotrusions on cicada wings which serves as a biomimetic model for the fabrication of antibacterial surfaces. Nanostructuring is obtained using various methodologies, for instance, sandblasting,^18,19^ electrospinning,^20,21^ non-thermal plasma treatment,^22^ electrochemical anodization,^14,23,24^ and hydrothermal method.^25,26^ The hydrothermal method is the most conventional, cost-effective, and scalable process for the surface modification of Ti and its alloys.^27^ In the case of materials that are used for medical applications, final surface treatment usually consists of sterilization. Thus, it is of great importance that the sterilization procedure does not alter nanotopography and by this does not influence the antibacterial properties of the surface. There lies a gap of knowledge related to the influence of different sterilization procedures on the nanostructured surfaces and their corresponding antibacterial effect.^28,29^ Nanostructured surfaces may significantly influence cellular and bacteria adhesion, proliferation, and differentiation,^23,30,31^ as well as the adhesion and activation of platelets,^13,32^ also due to their specific electric properties.^33,32^

The present work aims to study the effect of modified surface properties on Ti and its alloys, namely, Ti foil (0.1 mm), Ti_6_Al_4_V discs, and NiTi foil. The different nanoscale morphologies obtained on these surfaces using hydrothermal methods were assessed using scanning electron microscopy (SEM), X-ray photoelectron spectroscopy (XPS), and water contact angle (WCA) measurements. The fabricated nanostructures were sterilized using an autoclave and ultraviolet (UV) light. The antibacterial effect on Escherichia coli was studied using ISO 22196 standard along with SEM imaging. The goal was to evaluate the antibacterial efficacy of nanostructured Ti, Ti_6_Al_4_V, and NiTi substrates against *E. coli*.

## 2 Results and Discussion

**Figure 1:**
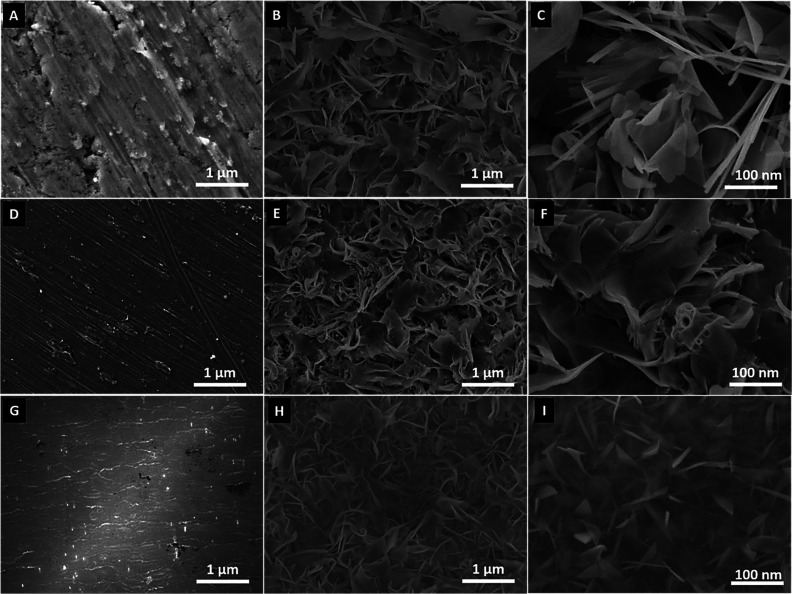
SEM images of untreated (A) Ti foil, (D) Ti_6_Al_4_V, (G) NiTi, and hydrothermally treated (B, C) Ti (Ti + HT), (E, F) Ti_6_Al_4_V, (Ti_6_Al_4_V + HT), and (H, I) NiTi (NiTi + HT).

The surface morphology of the as-received Ti, Ti_6_Al_4_V, and NiTi substrates and the hydrothermally (HT) treated substrates, i.e., Ti + HT, Ti_6_Al_4_V + HT and NiTi + HT was analyzed by SEM as shown in Figure 1. The SEM analysis of untreated samples in Figure 1A,D,G shows microstructured surface morphology, whereas in the case of hydrothermally treated surfaces, different morphologies were obtained. For the purpose of comparison, synthesis conditions were the same for all of the three samples (Ti, Ti_6_Al_4_V, and NiTi) (Section 4.2). The Ti + HT sample (Figure 1B,C) depicted a network consisting of a feather-like structure incorporated with elongated features at the nanoscale which could act as nanoprotrusions/needles. In the case of the Ti_6_Al_4_V + HT (Figure 1E,F) sample, a flaky feather-like compact structure can be observed, whereas for the NiTi + HT sample (Figure 1G,H), a nanograss-like morphology is observed on the surface. This shows that after hydrothermal treatment alterations in surface topography could be achieved, and morphology could be tailored by using the corresponding starting material.

From XPS analysis, it was confirmed that all samples have very similar surface chemistry, as basically only Ti, O, and C were detected on these surfaces (Table 1). Only in the case of NiTi about 2 atom % of Ni was also detected on the surface. It can be observed that after hydrothermal treatment, a decrease in C and an increase in O and Ti are detected for Ti and Ti_6_Al_4_V, with a more pronounced increase in oxygen for the case of Ti_6_Al_4_V. While the opposite is observed for NiTi as an increase in C and a decrease in O and Ti after HT treatment are observed. The concentration of Ni, however, remains practically unchanged. As in the case of NiTi, peaks corresponding to Ni or its oxides (peaks in the range of 850–860 eV)^34,35^ are observed on the surface even after HT treatment; this gives evidence that Ni from the substrate is involved in the chemical reaction during HT treatment.

**Table 1:** XPS Analysis of Ti, Ti_6_Al_4_V, and NiTi Surface and HT Treated Surfaces

atom %	C	O	Ti	Ni	O/Ti	Ni/Ti
Ti	52.3	36.7	11.0		3.34	
Ti + HT	31.3	48.7	20.0		2.43	
Ti_6_Al_4_V	28.6	54.9	16.5		3.33	
Ti_6_Al_4_V + HT	19.5	59.5	21.0		2.83	
NiTi	16.9	59.5	21.4	2.2	2.78	0.10
NiTi + HT	27.2	54.2	15.8	2.8	3.43	0.18

It has already been shown by other authors that Ni-depleted TiO_2_ on the surface of NiTi is hard to achieve.^36^ Wang et al.^37^ similarly synthesized TiO_2_ on the NiTi with the hydrothermal method in an NaOH solution, without Ti isopropoxide; however, the oxide formed contained about 20–30 atom % of Ni. Hang et al.^38^ hydrothermally treated NiTi in an ultrapure water at 200 °C and showed that 30 min HT treatment decreases the Ni/Ti ratio (in comparison the Ni/Ti ratio is 0.28 for the untreated sample) close to 0, while prolonged HT treatment time (i.e., 60 and 120 min) led to increased Ni content on the surface until the Ni/Ti ratio reached 1.0 after HT treatment for 240 min. However, in our case the Ni/Ti ratio for the untreated sample was 0.1 and for the HT-treated one it only slightly increased to 0.18.

**Figure 2:**
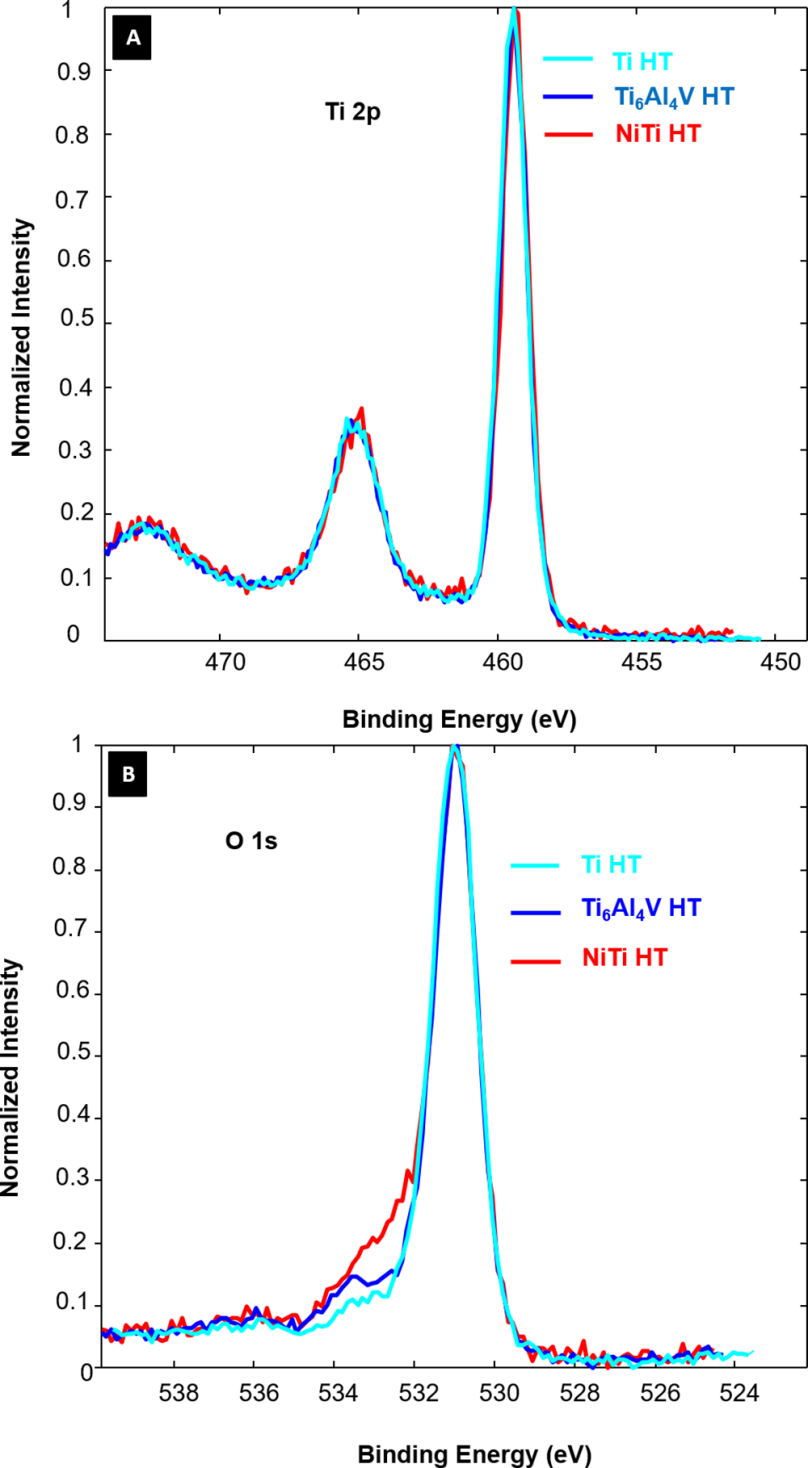
High-resolution spectra obtained from XPS analysis for (A) Ti 2p and (B) O 1s for Ti + HT, Ti_6_Al_4_V + HT, and NiTi + HT.

Interestingly, sodium was not detected by XPS on the hydrothermally treated surfaces, which confirms that Na ions are not incorporated into the oxide layer. In the case of Ti_6_Al_4_V, no V or Al was detected on the untreated or on the HT-treated surface, which gives evidence that uniform titanium oxide is formed on this surface. High-resolution spectra were also recorded, and it can be observed that all HT-treated samples have very similar Ti 2p peaks, which correspond to stoichiometric TiO_2_ on the surface as seen in Figure 2A. Small changes in O 1s spectra (Figure 2B) are observed mainly in the case of Ni + HT, where a more intense left shoulder peak is observed (binding energy between 532 and 533 eV), which corresponds to hydroxyl or chemisorbed water (OH_2_)^39^ or even NiO and NiTiO_3._^35^ In the case of Ti_6_Al_4_V, a slightly higher peak compared to Ti is also observed and could be correlated with a higher oxygen content, which could correspond to O–H bonds from hydroxyl groups or water molecules at the sample surface.

**Figure 3:**
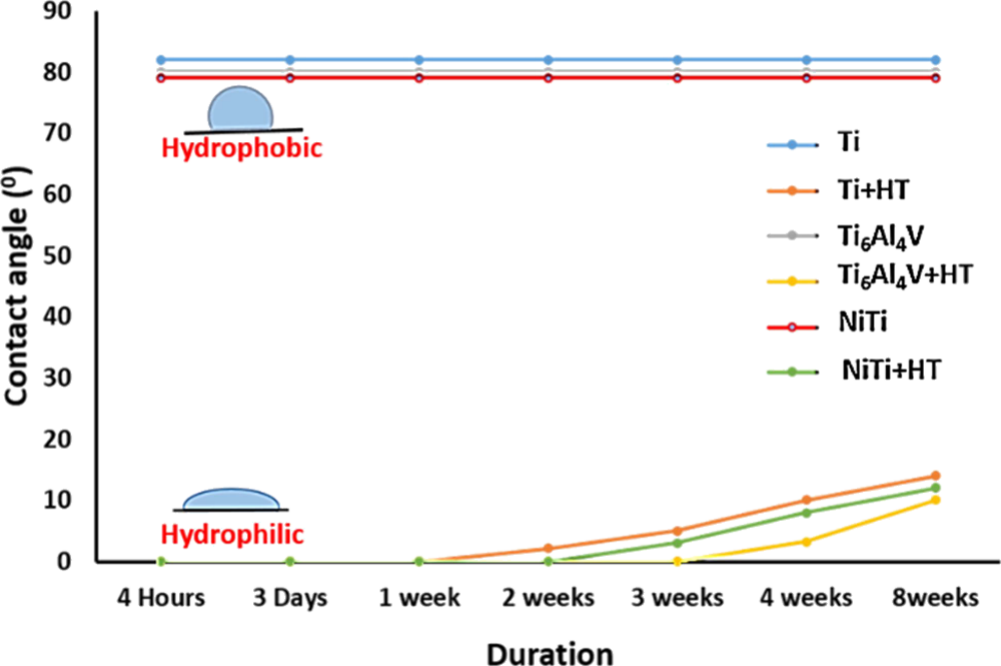
WCA of untreated and HT-treated samples over the duration of 8 weeks.

WCA measurements of untreated samples (Ti, Ti_6_Al_4_V, and NiTi) showed the hydrophobic character of surfaces (Figure 3). The WCA of untreated Ti, Ti_6_Al_4_V, and NiTi was 82, 80, and 79°, respectively. After the HT treatment, samples, however, become superhydrophilic; the WCA measured immediately after the hydrothermal synthesis (4 h) of Ti, Ti_6_Al_4_V, and NiTi was not measurable (less than 5°). The samples showed superhydrophilic properties even 1 week after storage in air; however, the WCA started to increase after 1, 2, or 3 weeks for Ti + HT, NiTi + HT, and Ti_6_Al_4_V + HT, respectively. The WCA measured 8 weeks after the HT synthesis is 14° for Ti + HT, 12° for NiTi + HT, and 10° for Ti_6_Al_4_V + HT. Thus, the HT-synthesized nanostructured surface exhibits hydrophilic nature even after 2 months of storage in air (WCA < 20°).

**Figure 4:**
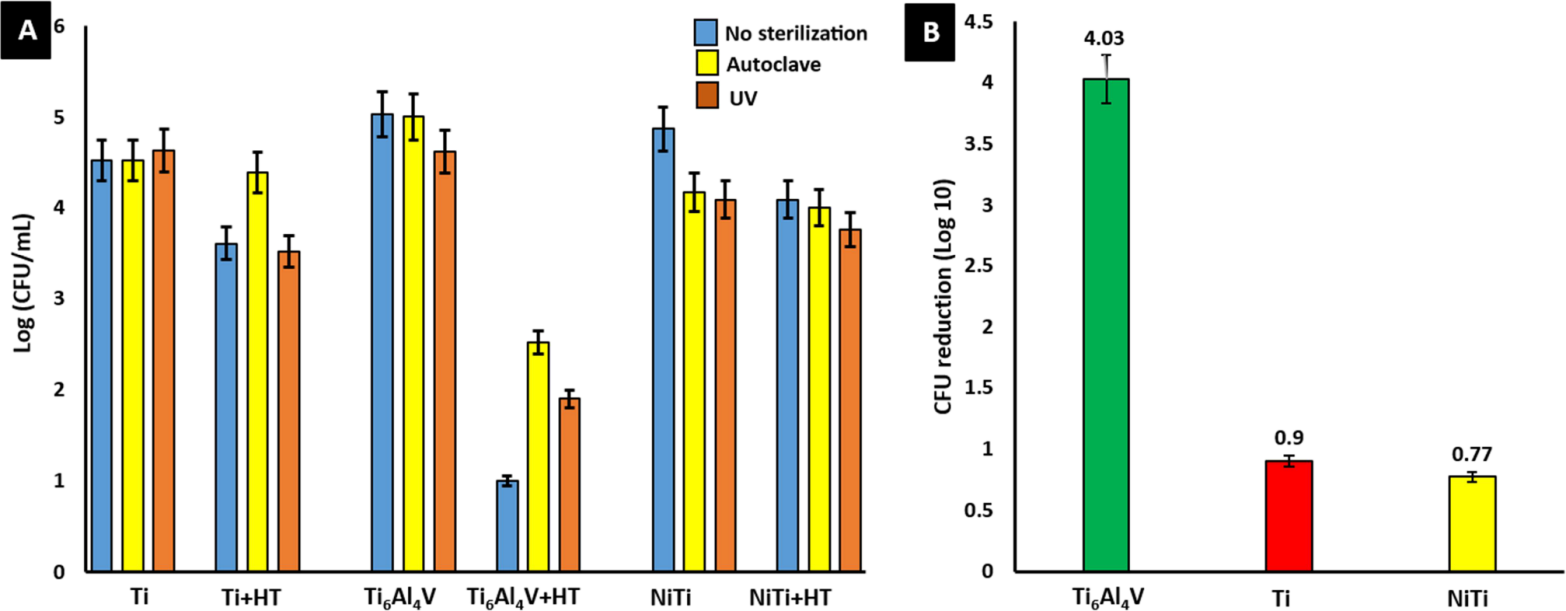
(A) Logarithmic calculation of *E. coli* CFU/mL on the autoclaved and UV-sterilized surface of the HT-treated and untreated samples, i.e., Ti, Ti + HT, Ti_6_Al_4_V, Ti_6_Al_4_V + HT, NiTi, and NiTi + HT, respectively. (B) Logarithmic reduction based on colony enumeration of HT treated Ti_6_Al_4_V, Ti, and NiTi in comparison with their control samples.

*E. coli* showed approximately 99% reduction in colony enumeration for HT-treated Ti_6_Al_4_V as compared to the control sample, whereas for HT-treated Ti and NiTi, the reduction in colony enumeration for *E. coli* growth was 87 and 83.3% respectively as compared to their control samples. For the purpose of comparison of different sterilization procedures, the antibacterial activity was determined for unsterilized samples, samples exposed to an autoclave, and samples sterilized by UV irradiation (see Section 4.4). It can be seen from Figure 4A that UV sterilization is better for reducing bacteria attachment compared to autoclave sterilization. However, this seemed not to be the case for Ti samples. In the case of untreated Ti, a similar amount of attached bacteria have been observed for nonsterilized and sterilized samples. For the HT-treated Ti, the UV sterilization did not alter the number of bacteria compared to unsterilized Ti + HT, while the autoclave seems to even increase bacterial attachment. In particular, the highest reduction of attached bacteria has been observed for hydrothermally treated Ti_6_Al_4_V, for which substantially lesser bacteria were observed on the surface. Figure 4B depicts logarithmic reduction of *E. coli* CFU/mL for HT-treated Ti, Ti_6_Al_4_V, and NiTi in comparison to their control samples.

**Figure 5:**
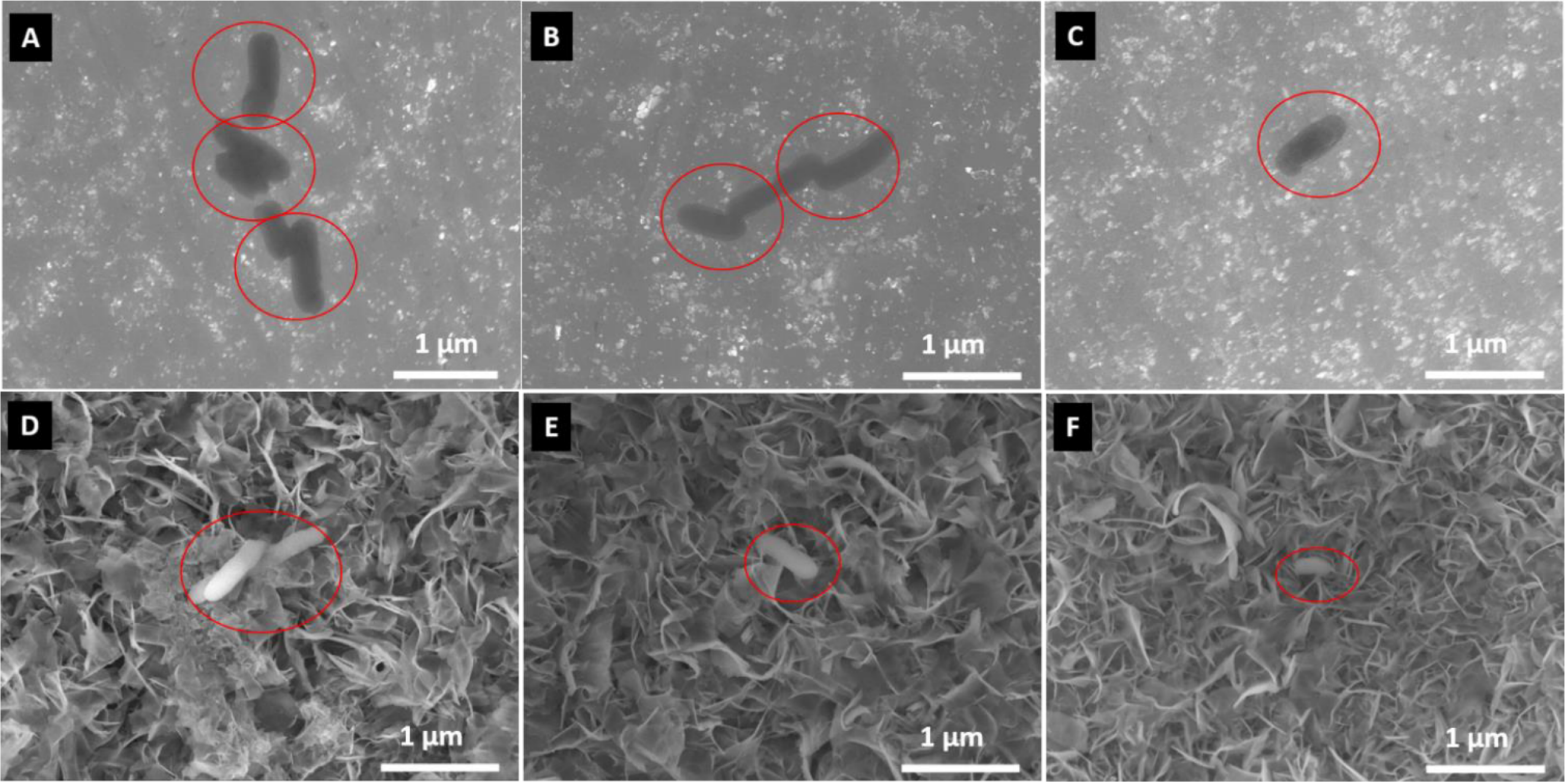
SEM images of *E. coli* after 24 h of incubation on untreated (A) Ti, (B) Ti_6_Al_4_V, (C) NiTi and HT treated samples (D), Ti + HT (E), Ti_6_Al_4_V + HT, and (F) NiTi + HT.

In Figure 5, the attached bacteria on the hydrothermally treated samples were analyzed by SEM. It can be observed that some bacteria are able to attach on the surface; however, damage of the membrane due to interaction with a rough surface is possible, which reduces their ability to colonize and form biofilms. From SEM images (Figure 5), it was confirmed that through the HT treatment using titanium tetraisopropoxide (TTIP) along with NaOH as an etchant, a surface topography could effectively be altered to the nanoscale. The type of morphology strongly depends on the initial substrate being used. Through recent studies, it has been established that upon altering the surface roughness and topography, bacterial attachment can be inhibited.^30^

**Figure 6:**
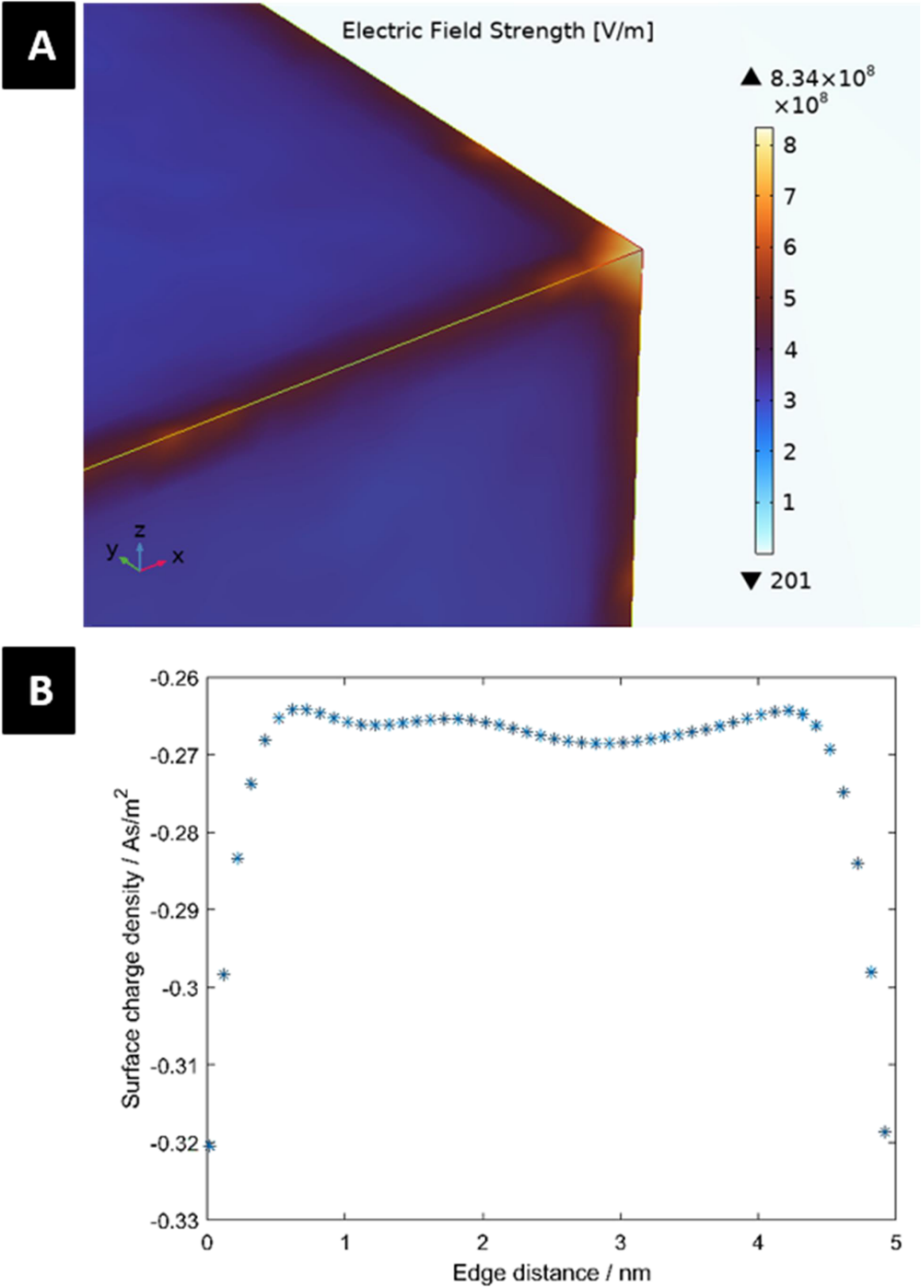
(A) Magnitude of the electrical field strength and (B) surface charge density along the sharp edges between two vertices. The values of the model parameters are a bulk electrolyte concentration of 150 mmoL/L and an electric potential of 110 mV.

Hydrothermally treated nanostructured surfaces, as prepared in this work, may influence the adhesion of bacteria also due to their specific electrostatic properties which are the consequence of many (convex) sharp edges and spicules/vertices as their important topological characteristic (see Figure 1). The electric field on the charged 3-D surface in contact with electrolyte solution was calculated within the modified Langevin–Poisson–Boltzmann model^42−44^ and numerically computed using COMSOL Multiphysics 6.0. The condition of constant electric potential on the surface was imposed in the numerical calculations. We can see that the magnitude of the surface charge density of (convex) sharp edges and vertices is increased (Figure 6B). Hence, in the vicinity of the surface sharp edges and spikes/vertices, the electric field strength is also increased, as shown in Figure 6A. As a result, the direct or mediated electrostatic interactions between the bacteria surface and the nanostructured substrate may be modified.^23,30,31,13,32^ In addition, due to the changes in physical topography, nanostructured surfaces display a bactericidal effect as the bacterial cell membrane is stretched leading to cell damage.^30^ Hasan and co-workers^40^ recently studied sharp spike-like nanostructures fabricated via HT etching for their effect against dental pathogens. They discussed that membrane perturbation or penetration at the point of contact tip between the cell membrane and nanostructure tip could eliminate bacteria in anaerobic conditions for both single-species (up to ∼94% cell death) and dual-species (up to ∼70% cell death). Ivanova et al.^41^ were the first to postulate the theory of mechano-bactericidal action of nanostructures by studying the nanopillar structure present on the surface of cicada wings and dragonfly for bactericidal properties.

They further showed that through mechanical action, conical nanopillars present on the surface of cicada wings cause lysis of the bacterial cell wall. Similarly, Linklater et al.^17^ proposed that nanopillars induce stretching of the cell membrane beyond its elasticity which results in membrane rupture. Also, nanoedges may extract the lipids and activate the reorientation of the lipid tails of the phospholipid bilayer leading to cell death and pore formation. The above-mentioned mechano-bactericidal action may have played a significant role in reduced bacterial adhesion observed on our fabricated HT-treated Ti, Ti_6_Al_4_V, and NiTi surfaces. Control samples had non-uniform plane morphology which turned into a rough nanoscale structure consisting of ridges along the flaky pattern observed after HT treatment for Ti_6_Al_4_V and NiTi. Recently, computational results^45^ have confirmed that nanostructured surfaces are capable of mechanically lysing bacteria. Among the three samples being studied here, Ti_6_Al_4_V + HT shows exceptional results as compared to Ti + HT and NiTi + HT with highest reduction in CFU/mL of *E. coli*. This could be due to the surface chemistry as Ti_6_Al_4_V + HT contains 59.5 atom % of O as compared to 48.7 and 54.2 atom % for Ti + HTi and NiTi + HT, respectively. It has been reported that bacterial attachment can be inhibited due to the reduced contact between the surface and cell.^46^ The mechanism involves trapping O in the substrate so that bacteria detach because of the limited contact area. Additionally, bacteria can also be trapped inside trenches, pits, cervices, or zones at the surface.^47^ This possibly explains the reason behind better results for Ti_6_Al_4_V + HT as compared to Ti + HTi and NiTi + HT as it contains more Ti and O on the surface. The difference in the thickness of the coating and its mechanical properties could also influence and should be further studied.

## 3 Conclusions

To summarize, nanostructured surfaces were prepared by hydrothermal treatment of Ti-based substrates (Ti, Ti_6_Al_4_V, and NiTi). The hydrothermal synthesis conditions were the same for all the samples. Therefore, the obtained morphology depends on the initial substrate being used. For Ti + HT, a feather-like nanostructure containing sharp protrusions was fabricated. For Ti_6_Al_4_V + HT, a nanofeather-like compact structure was obtained, whereas for NiTi + HT nanograss-like morphology was obtained. XPS analysis showed that surface chemistry is also substrate-dependent; on the Ti and Ti_6_Al_4_V, pure TiO_2_ is formed after the hydrothermal synthesis, while the TiO_2_ layer on the NiTi contains a small amount of Ni. The presence of Ni could be problematic when used in materials for medical applications, such as implants, since Ni can corrode and be released into the body. This can be an issue when NiTi is used as an implant material. Antibacterial activity tests showed that the hydrothermally treated surfaces (Ti + HT, Ti_6_Al_4_V + HT, and NiTi + HT) possess higher antibacterial activity compared to untreated control samples. This could be due to altered morphology; nanofeatures on the surface of hydrothermally treated samples can mechanically rupture the bacterial cells. Basically, all UV-treated surfaces, except Ti_6_Al_4_V + HT, exhibited better antibacterial properties. This could be due to the formation of reactive oxygen species by the photocatalytic effect^48^ of the TiO_2_ surface and could additionally promote the bacterial killing mechanism. However, the highest antibacterial activity was confirmed for the Ti_6_Al_4_V + HT surface, which was chemically and morphologically very similar to Ti + HT. The main observed difference was only in the concentration of oxygen on the surface, which could be present also in O–H bonds or water molecules at the surface. In conclusion, the effect of surface properties (morphology, elemental composition, wettability) of materials commonly used in biomedical applications (Ti, Ti_6_Al_4_V, and NiTi) on the bacterial activity has been shown. Further studies of mechanical properties, ion-release, and corrosion resistance, as well as interactions with human cells, are necessary to perform on these materials in order to obtain detailed information about biocompatibility.

## 4 Experimental Methods

### 4.1 Sample Preparation

Ti foil (thickness: 0.10 mm, Advent, 99.6+%), Ti_6_Al_4_V discs (Tifast S.R.I., titanium grade 23Ti_6_Al_4_V ELI), and NiTi foil (thickness: 0.38 mm, Alfa Aesar, flat annealed, pickled surface) were washed in acetone, ethanol (96% and absolute, Sigma-Aldrich), and water (milliQ, Merck) for 10 min respectively inside the beaker and subjected to ultrasound. Afterward, the samples were dried at 70 °C in a furnace on an Al-crucible (app. 1 h).

### 4.2 Synthesis

An aqueous solution (30 mL) containing 2 mL of titanium(IV) isopropoxide (97%, Sigma-Aldrich) was prepared using deionized H_2_O (milliQ, Merck) and NaOH (reagent grade, 90%, flakes, Sigma-Aldrich) to adjust the pH of the solution to 13. Thereafter, the prepared aqueous solution of titanium isopropoxide was poured onto the cleaned and dried samples kept inside a Teflon vessel. This Teflon vessel was sealed inside a stainless-steel reactor and kept inside a furnace at a temperature of 200 °C for 24 h which was then cooled to room temperature. Samples were vigorously washed with deionized H_2_O and ultrasonicated for 3 min. Later samples were dried in a furnace for 2 h at 70 °C and then cooled to room temperature inside the fume hood.

### 4.3 Antibacterial Test

The pathogenic strain of *E. coli* was first prepared in Luria–Bertani broth for 24 h at 37 °C (*Escherichia coli*, Strain K12, lyophilized cells, Sigma-Aldrich). A suspension of *E. coli* (10^5^ colony forming unit (CFU/mL) was prepared, from which 0.1 mL was pipetted onto the sample surfaces. The samples were then incubated in the incubator (I-105 CK UV, Kambič) for 24 h at 37 °C in a humidity box in order to maintain relative humidity at 90%. After incubation, *E. coli* on the surface was removed using 2.5 mL of sterilized phosphate buffered saline (PBS - tablets, Sigma-Aldrich), and 0.2 mL of this solution was taken for inoculation of *E. coli* in the Nutrient agar plate at 37 °C for 24 h. Then the number of CFUs can be determined. For convenient counting of CFUs, before inoculating *E. coli* in the Nutrient agar plate, the initial solution was diluted further with PBS by a factor of 10^0^–10^5^. The CFU/mL was calculated using an automated colony counter (Acolyte 3, Synbiosis).

### 4.4 Sterilization

The autoclave sterilization of samples was carried out in Autoclave A-21CA, Kambič for 15 min in dry mode. UV irradiation of samples was performed with UV-C light (Sylvania ultraviolet G15W; 15 W/cm^2^) for 15 min.

### 4.5 Characterization

#### 4.5.1 Scanning Electron Microscopy

The morphological analysis of the materials was conducted using a scanning electron microscope (JEOL JSM-7600F) at an accelerating voltage of 5 kV. The test was done in triplicate, and only representative images are shown.

#### 4.5.2 WCA Analysis

The surface wettability was performed with Drop Shape Analyzer DSA-100 (Krüss GmbH, Hannover, Germany) by a sessile drop method to measure a static contact angle. The contact angle on the surface was analyzed on freshly prepared samples and on untreated samples, which were prior to analysis cleaned with acetone, ethanol, and d.H_2_O respectively for 5 min each via ultrasonication. A 2.5 μL drop of deionized water (MilliQ, Merck) was put on eight different areas of the surface, and the average value was calculated. The relative humidity was around 45% and the operating temperature was 21 °C, which did not vary significantly during continuous measurements, nor after storage. The contact angle was measured for HT-treated fresh samples (4 h) and later during the course of 3 days, 1 week and continued till 8 weeks stored in closed containers.

#### 4.5.3 X-ray Photoelectron Spectroscopy

The XPS analyses were carried out on the PHI-TFA XPS spectrometer produced by Physical Electronics Inc. Samples were put on the sample holder and were introduced into the ultra-high vacuum spectrometer. The analyzed area was 0.4 mm in diameter, and the analyzed depth was about 3–5 nm. Sample surfaces were excited by X-ray radiation from a monochromatic Al source at a photon energy of 1486.6 eV. Three different XPS measurements were performed on each sample, and the average composition was calculated.

## Abbreviations

IAI: implant-associated infections
HT: hydrothermal method
